# The Role of Motivation in Complex Problem Solving

**DOI:** 10.3389/fpsyg.2017.00851

**Published:** 2017-05-23

**Authors:** C. Dominik Güss, Madison Lee Burger, Dietrich Dörner

**Affiliations:** ^1^Department of Psychology, University of North FloridaJacksonville, FL, United States; ^2^Trimberg Research Academy, University of BambergBamberg, Germany

**Keywords:** complex problem solving, dynamic decision making, simulation, motivation, PSI-theory, Maslow's hierarchy of needs, achievement motivation

## The role of motivation in complex problem solving

Previous research on Complex Problem Solving (CPS) has primarily focused on cognitive factors as outlined below. The current paper discusses the role of motivation during CPS and argues that motivation, emotion, and cognition interact and cannot be studied in an isolated manner. Motivation is the process that determines the energization and direction of behavior (Heckhausen, 1991).

Three motivation theories and their relation to CPS are examined: McClelland's achievement motivation, Maslow's hierarchy of needs, and Dörner's needs as outlined in PSI-theory. We chose these three theories for several reasons. First, space forces us to be selective. Second, the three theories are among the most prominent motivational theories. Finally, they are need theories postulating several motivations and not just one. A thinking-aloud protocol is provided to illustrate the role of motivational and cognitive dynamics in CPS.

Problems are part of all the domains of human life. The field of CPS investigates problems that are complex, dynamic, and non-transparent (Dörner, 1996). Complex problems consist of many interactively interrelated variables. Dynamic ones change and develop further over time, regardless of whether the involved people take action. And non-transparent problems have many aspects of the problem situation that are unclear or unknown to the involved people.

CPS researchers focus exactly on such kinds of problems. Under a narrow perspective, CPS can be defined as thinking that aims to overcome barriers and to reach goals in situations that are complex, dynamic, and non-transparent (Frensch and Funke, 1995). Indeed, past research has shown the influential role of task properties (Berry and Broadbent, 1984; Funke, 1985) and of cognitive factors on CPS strategies and performance, such as intelligence (e.g., Süß, 2001; Stadler et al., 2015), domain-specific knowledge (e.g., Wenke et al., 2005), cognitive biases and errors (e.g., Dörner, 1996; Güss et al., 2015), or self-reflection (e.g., Donovan et al., 2015).

Under a broader perspective, CPS can be defined as the study of cognitive, emotional, motivational, and social processes when people are confronted with such complex, dynamic and non-transparent problem situations (Schoppek and Putz-Osterloh, 2003; Dörner and Güss, 2011, 2013; Funke, 2012). The assumption here is that focusing solely on cognitive processes reveals an incomplete picture or an inaccurate one.

To study CPS, researchers have often used computer-simulated problem scenarios also called microworlds or virtual environments or strategy games. In these situations, participants are confronted with a complex problem simulated on the computer from which they gather information, and identify solutions. These decisions are then implemented into the system and result in changes to the problem situation.

## Previous research on motivation and CPS

The idea to study the interaction of motivation, emotion, and cognition is not new (Simon, 1967). However, in practice this has been rarely examined in the field of CPS. One study assessed the need for cognition (i.e., the tendency to engage in thinking and reflecting) and showed how high need of cognition was related to broader information collection and better performance in a management simulation (Nair and Ramnarayan, 2000).

Vollmeyer and Rheinberg (1999, 2000) explored in two studies the role of motivational factors in CPS. They assessed mastery confidence (similar to self-efficacy), incompetence fear, interest, and challenge as motivational factors. Their results demonstrated that mastery confidence and incompetence fear were good predictors for learning and for knowledge acquisition.

## CPS assessment

Before we describe three theories of motivation and how they might be related and applicable to CPS, we will briefly describe the WINFIRE computer simulation (Gerdes et al., 1993; Schaub, 2009) and provide a part of a thinking-aloud protocol of one participant while working on WINFIRE. WINFIRE is the simulation of small cities surrounded by forests. Participants take the role of fire-fighting commanders who try to protect cities and forests from approaching fires. Participants can give a series of commands to several fire trucks and helicopters. In WINFIRE quick decisions and multitasking are required in order to avoid fires spreading. In one study, participants were also instructed to think aloud, i.e., to say aloud everything that went through their minds while working on WINFIRE. These thinking-aloud protocols, also called verbal protocols, were audiotaped and transcribed in five countries and compared (see Güss et al., 2010).

The following is a verbatim WINFIRE thinking-aloud protocol of a US participant (Güss et al., 2010):

Ok, I don't see any fires yet. I'm trying to figure out how the helicopters pick up the water from the ponds. I put helicopters on patrol mode. Not really sure what that does. It doesn't seem to be moving. Oh, there it goes, it's moving…I guess you have to wait till there's a fire showing…Ok, fire just started in the middle, so I have to get some people to extinguish it. Ok, now I have another fire going here. I'm in trouble here. Ok. Ok, when I click extinguish, it don't seem to respond. Guess I'm not clear how to get trucks right to the fire. Ok, one fire has been extinguished, but a new one started in the same area. I'm getting more trucks out there trying to figure out, how to get helicopters to the pond. I still haven't figured that out, because they have to pick up the water. Ok, got a pretty good fire going here, so I'm going to put all the trucks on action, ok, water thing is making me mad. Ok. I'm not sure how it goes? Ok, the forest is burning up now—extinguish! Ok, ok, I'm in big trouble here…

## Psychological theories of motivation and their application to CPS

### Mcclelland's human motivation theory

In his Human Motivation theory, McClelland distinguishes three needs (power, affiliation, and achievement) and argues that human motivation is a response to changes in affective states. A specific situation will cause a change in the affective state through the non-specific response of the autonomic nervous system. This response will motivate a person toward a goal to reach a different affective state (McClelland et al., 1953). An affective state may either be positive or negative, determining the direction of motivated behavior as either approach oriented, i.e., to maintain the state, or avoidance oriented, i.e., to avoid or discontinue the state (McClelland et al., 1953).

Motivation intensity varies among individuals based on perception of the stimulus and the adaptive abilities of the individual. Hence, when a discrepancy exists between expectation and perception, then a person will be motivated to eliminate this discrepancy (McClelland et al., 1953). In the statement from the thinking-aloud protocol we can infer the participant's achievement motivation, “*Guess I'm not clear how to get trucks right to the fire. Ok, one fire has been extinguished, but a new one started in the same area.”* The participant at first begins to give up and reduce effort, but then achieves a step toward the goal. This achievement causes the reevaluation of the discrepancy between ability and the goal as not too large to overcome. This realization motivates the participant to continue working through the scenario. Whereas, the need for achievement seems to guide CPS, the needs for power and affiliation cannot be observed in the current thinking-aloud protocol.

Based on the previous discussion we can derive the following predictions:

*Prediction 1*: Approach-orientation will lead to greater engagement in CPS compared to avoidance-orientation.
*Prediction 2*: Based on an individual's experience either power, affiliation, or achievement will become dominant and guide the strategic approach in CPS.

### Maslow's hierarchy of needs

Maslow's Hierarchy of Needs (Maslow, 1943, 1954) suggests that everyone has five basic needs that act as motivating forces in a person's life. Maslow's hierarchy takes the form of a pyramid in which needs lower in the pyramid are primary motivators. They have to be met before higher needs can become motivating forces. At the bottom of the pyramid are the most basic needs beginning with physiological needs, such as hunger, and followed by safety needs. Then follow the psychological needs of belongingness and love, and then esteem. Once these four groups of needs have been met, a person may reach the self-fulfillment stage of self-actualization at which time a person can be motivated to achieve ones full potential (Maslow, 1943).

The first four groups of needs are external motivators because they motivate through both deficiency and fulfillment. In essence, a person fulfills a need which then releases the next unsatisfied need to be the dominant motivator (Maslow, 1943, 1954). The safety need is often understood as seeking shelter, but Maslow also understands safety also as wanting “a predictable, orderly world” (Maslow, 1943, p. 377), “an organized world rather than an unorganized or unstructured one” (Maslow, 1943, p. 377). Safety refers to the “common preference for familiar rather than unfamiliar things” (Maslow, 1943, p. 379).

In this sense the safety need becomes active when the person does not understand what is happening in the microworld, as the following passage of the thinking-aloud protocol illustrates. “*I put helicopters on patrol mode. Not really sure what that does. It doesn't seem to be moving.”* The safety need is demonstrated in the person's desire for organization, since unknown and unexpected events are seen as threats to safety.

The esteem need as a motivator becomes evident through the statement, “*Guess I'm not clear how to get trucks right to the fire.”* The participant becomes aware of his inability to control the situation which affects his self-esteem. The esteem need is never fulfilled in the described situation and remains the primary motivator. The following statements show how affected the participant's esteem need is by the inability to control the burning fires. “*Ok. I'm not sure how it goes? Ok, the forest is burning up now—extinguish! Ok, ok, I'm in big trouble*.”

*Prediction 3*: A strong safety need will be related to elaborate and detailed information collection in CPS compared to low safety need.
*Prediction 4*: People with high esteem needs will be affected more by difficulties in CPS and engage more often in behaviors to protect their esteem compared to people with low esteem needs.

### Dörner's theory of motivation as part of PSI-theory

PSI-theory described the interaction of cognitive, emotional, and motivational processes (Dörner, 2003; Dörner and Güss, 2011). Only a small part of the theory is examined here. Briefly, the theory encompasses five basic human needs: the existential needs (thirst, hunger, and pain avoidance), the sexuality need, and the social need for affiliation (group binding), the need for certainty (predictability), and the need for competence (mastery). If the environment is unpredictable, the certainty need becomes active. If we are not able to cope with problems, the competence need becomes active. The need for competence also becomes active when any other need becomes activated. With an increase in needs, the arousal increases.

The first three needs cannot be observed or inferred from the thinking-aloud protocol provided. Statements like, “I'm trying to figure out how the helicopters pick up the water from the ponds.” and “Guess I'm not clear how to get trucks right to the fire,” demonstrate the needs for certainty and competence, i.e., to make the environment predictable and controllable.

The following statements reflect the participant's need for competence, i.e., the inefficacy or incapability of coping with problems. “*I'm in trouble here…ok, water thing is making me mad*.” Not being able to extinguish the fires that are approaching cities and are destroying forests is experienced as anger. The arousal rises as the resolution level of thinking decreases. So, the participant does not think about different options in an elaborate manner. Yet, the participant becomes aware of his failure. The competence need then causes the participant to search for possible solutions, “*I still haven't figured that out because they have to pick up the water…”* The need for competence is satisfied when the problem solver is able to change either the environment or ones views of the environment.

*Prediction 5*: A strong certainty need is positively related to a strong competence need.
*Prediction 6*: High need for certainty paired with high need for competence can lead to safeguarding behavior, i.e., background monitoring.
*Prediction 7*: An increase in the competence and uncertainty needs leads to increased arousal and a lower resolution level of thinking. CPS becomes one-dimensional and possible long-term and side-effects are not considered adequately.

### Summary and evaluation

We have briefly discussed three motivation theories and their relation to CPS referring to one thinking-aloud protocol: McClelland's achievement motivation, Maslow's hierarchy of needs, and Dörner's needs as outlined in PSI-theory.

A Comparison of Three Need Theories in the Context of CPS.

**Table:** 

	**McClelland's achievement motivation**	**Maslow's hierarchy of need**	**Dörner's theory of motivation as part of PSI-theory**
Scope/ Breadth	+	++	++
Applicability to CPS	+	+	++
Adaptability of needs	−	−	+
Incorporation of emotion	++	−	++
Individual differences	++	−	++
*Evaluation criteria: very small/very low −, small/low −, much/high +, very much/very high ++*.			

Comparing the scope of the three theories and referring to the scope and different needs covered in the three theories, McClelland's theory describes three needs (power, affiliation, and achievement), Maslow's theory describes five groups of needs (physiological, safety, love and belonging, esteem, self-actualization), and Dörner's theory describes five different needs (existential, sexuality, affiliation, certainty, and competence).

All three theories can be applied to CPS. McClelland's need for achievement, Maslow's needs for esteem and safety, and Dörner's needs for certainty and competence could be inferred from the thinking–aloud passage. The need for affiliation which is a part of each of the three theories could play an important role when groups solve complex problems.

The existential needs and the need for affiliation outlined in PSI-theory can also be found in Maslow's hierarchy of needs. These two theories differ in the adaptability of the needs. However, Maslow's esteem needs are only activated as the primary motivator as the physiological needs, belongingness, and love needs are met. The needs are more fluidly described as motivators in PSI-theory. One need becomes the dominant motive according to the expectancy–value principle. Expectancy stands for the estimated likelihood of success. The value of a motive stands for the strength of the need. According to McClelland's theory, the role of three motivations develops through life experience in a specific culture; and often times, one of the three becomes the main driving force for a person, almost like a personality trait. In that sense, there is not much flexibility.

Motivation and emotion are closely related as became partially clear in the discussion of McClelland's theory. Emotions are discussed in detail in PSI-theory, but space does not allow us to discuss those in detail here (see Dörner, 2003). Emotions are not described in detail in Maslow's Hierarchy of Needs.

Individual differences in motivation and needs are discussed in two of the three theories. According to McClelland, a person develops an individual achievement motive by learning one's own abilities from past achievements and failures. Based on different learning histories, different persons will have a different dominant motivation guiding behavior in a given situation. Learning history also influences the competence need in PSI-theory. Additionally PSI-theory assumes individual differences that are simulated through different individual motivational parameters in the theory. The certainty need, for example, becomes active when there is a deviation from a given set point. Individual differences are related to different set points and how sensitive the deviations are (e.g., deviation starts quickly vs. deviation starts slowly).

## Conclusion

The thinking-aloud example from the WINFIRE microworld described earlier demonstrates that a person's CPS process is influenced by the person's needs. We have focused in our discussion on motivational processes that are considered in the framework of need theories. Beyond that, other motivational theories exist that focus on the importance of motivation for learning and achievement (e.g., expectancy, reasons for engagement, see Eccles and Wigfield, 2002). Thus, the applicability of these theories to CPS could be explored in future studies as well.

We discussed the three motivational theories of McClelland's Achievement Motivation, Maslow's Hierarchy of Need, and Dörner's Theory of Motivation as part of PSI-Theory. Although, the theories differ our discussion has shown that the three theories can be applied to CPS. Problem solving is a motivated process and determined by human motivations and needs.

## Author contributions

The first author CG conceptualized the manuscript, selected the thinking-aloud passage, the second author MB primarily summarized McClellands and Maslow's theories. All authors contributed to writing up the manuscript.

### Conflict of interest statement

The authors declare that the research was conducted in the absence of any commercial or financial relationships that could be construed as a potential conflict of interest.
